# Diagnosis and Treatment of Esophageal Candidiasis: Current Updates

**DOI:** 10.1155/2019/3585136

**Published:** 2019-10-20

**Authors:** Abdimajid Ahmed Mohamed, Xin-liang Lu, Faycal Awaleh Mounmin

**Affiliations:** Department of Gastroenterology, The Second Affiliated Hospital of Zhejiang University, Hangzhou, China

## Abstract

Esophageal candidiasis (EC) is the most common type of infectious esophagitis. In the gastrointestinal tract, the esophagus is the second most susceptible to candida infection, only after the oropharynx. Immunocompromised patients are most at risk, including patients with HIV/AIDS, leukemia, diabetics, and those who are receiving corticosteroids, radiation, and chemotherapy. Another group includes those who used antibiotics frequently and those who have esophageal motility disorder (cardiac achalasia and scleroderma). Patients complained of pain on swallowing, difficulty swallowing, and pain behind the sternum. On physical examination, there is a plaque that often occurs together with oral thrush. Endoscopic examination is the best approach to diagnose this disease by directly observing the white mucosal plaque-like lesions and exudates adherent to the mucosa. These adherent lesions cannot be washed off with water from irrigation. This disease is confirmed histologically by taking the biopsy or brushings of yeast and pseudohyphae invading mucosal cells. The treatment is by systemic antifungal drugs given orally in a defined course. It is important to differentiate esophageal candidiasis from other forms of infectious esophagitis such as cytomegalovirus, herpes simplex virus, gastroesophageal reflux disease, medication-induced esophagitis, radiation-induced esophageal injury, and inflammatory conditions such as eosinophilic esophagitis. Except for a few complications such as necrotizing esophageal candidiasis, fistula, and sepsis, the prognosis of esophageal candidiasis has been good.

## 1. Introduction

Candida is a yeast organism that colonizes the surface epithelium of the alimentary canal and urogenital system of healthy human beings as normal flora. When there is an impaired local or systemic immune system, candida overgrowth may occur, leading to candida infection. More than 15 distinct candida species can cause diseases, and the most common pathogens are *C. albicans*, *C. glabrata* and *C. tropicalis* [1]. The pathogenicity of these pathogens varies from species to species, and so does the degree of damage to the immune system. Mucosal candida infections, especially those involving oropharynx, esophagus, and vagina, are most common in the general population. The most common cause of infectious esophagitis is candida infection of the esophagus, with an incidence of up to 88% [2, 3]. Normally, candida is a symbiont of the esophagus. When host defense mechanisms are impaired, it allows candida to proliferate in esophageal mucosa and form adhesive plaques [4].

Esophageal candidiasis (EC) is usually common among patients infected with human immunodeficiency virus (HIV). This is because approximately 10–15% of HIV-infected patients will develop EC [5–7] during their lifetime, while another 85–90% of HIV-infected patients will develop oropharyngeal candidiasis [8]. We have found that the incidence of EC was 0.32% in individuals with strong immunity in Korea in a single-center study [9].

## 2. Etiology

The occurrence of infection is the result of the interaction between pathogen and host, especially related to the immune status of the body and whether the patient has basic diseases. The diagnosis of fungal esophagitis was first presented in 1839, and candida was identified as the pathogen. Under normal circumstances, both the specific defense system and the nonspecific defense system of the body's digestive tract can inhibit the excessive growth of fungi [4]. After the functional deficiency of host immune system or the application of antibiotics, the composition of microflora in the digestive tract changes, and the invasion ability of opportunistic pathogenic fungi is enhanced through the gene regulation mechanism, leading to opportunistic fungal infection [5]. Candida is one of the common opportunistic pathogenic fungi. The pathogenicity of candida may be related to its morphology, adhesion to tissues, and production of extracellular proteases. Furthermore, the destruction of local defense mechanism and systemic factors including low immune function, unreasonable application of antibiotics and hormones, physiological weakness, endocrine disorder, nutritional factors, chemotherapy, radiotherapy, and the presence of malignant diseases may contribute to the occurrence of this disease.

## 3. Risk Factors

Several studies have shown that the incidence of esophageal candidiasis is 0.32% to 5.2% in the general population. But, there are some specific populations in which the incidence of this disease is higher, while others are low. This paper attempts to assess risk factors from the following aspects.

### 3.1. Gender

Esophageal candidiasis affects all patients irrespective of gender. For example, a study conducted by Nassar et al. on individuals with this disease who were immunocompetent showed that there was no difference in terms of gender [10].

### 3.2. Age

Worldwide, the median age of patients with esophageal candidiasis is 55.5 years. In the recent study, Kliemann et al. reported that the age range of esophageal candida disease patients was 21–88 years old (average 57.4 years old; standard deviation 16.7 years) [2]. However, other factors, such as the use of medications, can also contribute to changes in the average age at which the disease occurs. Therefore, the disease may occur at early ages or late. The average age of the patients at the time of diagnosis was 39.8 years [10].

### 3.3. Comorbidities

Approximately 10% of HIV patients develop esophageal candidiasis in their lifetime [8]. However, the trend of this infection among HIV-positive patients is decreasing because of the effectiveness of highly active antiretroviral therapy (HARRT) [11]. In the present age, there is a rise in several cases in non-HIV patients, possibly because of comorbidities such as diabetes mellitus, peptic ulcer diseases [12], or medications such as antibiotics and corticosteroids given to patients who received transplant organs [1]. In addition, the condition called cardiac achalasia, a motor disorder of the esophagus, may cause stasis of food and secretions in the esophagus, which leads to overgrowth of *Candida albicans* and development of esophageal candida infections [13, 14].

### 3.4. Use of Proton-Pump Inhibitors

This is the most common cause of CE in individuals with strong immunity. In fact, about 72% of HIV-negative patients used proton-pump inhibitors (PPI) [15] and other acid suppression drugs. Hoversten et al. reported that PPI was the most common risk in individuals with strong immunity, contributing 63%–81% to the occurrence of candida esophagitis [15].

### 3.5. Smoking

Some studies suggest that smoking is also associated with the development of esophageal candidiasis. Firstly, the presence of chemicals weakens the local immune surface of esophageal squamous epithelium. Subsequently, symbiotic bacteria such as *Candida albicans* were allowed to invade and proliferate, leading to candida esophagitis [1, 12].

## 4. Pathophysiology

The mucous membrane of the esophagus is naturally lined by the protective innate immune mechanical barrier called the nonkeratinized stratified squamous epithelium. Because of this, *Candida albicans* may be part of the commensal that colonizes the esophagus in some individuals, accounting for about 20% [16]. However, processes that impair the immune system, as well as those that cause local lesions in the esophageal upper cortex, can lead to the proliferation and colonization of *Candida albicans*. Subsequently, candida adheres to the mucous membrane and forms yellow-white patches. We can see the plaques on upper endoscopy and cannot wash from the mucosa with water irrigation. These plaques can be found diffusely throughout the entire esophagus or localized in the upper, middle, or distal esophagus [11].

## 5. Management of Candida Esophagitis

### 5.1. History and Physical Examination

The clinical manifestations of the patients are often related to the extent of esophageal mucosal damage, and the most common symptoms are pain on swallowing, difficulty swallowing, and pain behind the sternum. Other symptoms include abdominal pain, heartburn, weight loss, diarrhea, nausea, vomiting, and melena [11, 17]. Esophageal endoscopic examination showed small white spots in the esophageal mucosa, and X-ray barium examination showed abnormal peristalsis at the upper and lower end of the esophagus. Only 15% of the patients show esophageal mucosal damage. Candida esophagitis can be divided into the following: (1) *acute infection*: extremely weak immunosuppression patients often die of acute fungal infection; (2) *subacute infection*: subacute infection may result in esophageal stricture or pseudodiverticulum; (3) *chronic infection*: usually from childhood, chronic infection is often associated with submucosal fungal infection and immunodeficiency.

### 5.2. Diagnosis

Because candida is a normal mycotic flora in the oral and gastrointestinal tract, isolation of candida from sputum and stool specimens cannot make a diagnosis of candida infection, which often requires histopathological evidence. The pathologic features of the endoscopic biopsy tissue are multiple abscesses with acute inflammatory reaction. Neutrophils are predominant, and fungal spores and pseudohyphae are visible.

If patients show typical clinical manifestations, candida is found in microbial cultures, and furthermore, there are high risk factors (such as broad-spectrum antibacterial drugs, corticosteroids, and immunosuppressive, and in intensive care unit, merge blood system basic diseases such as tumor, diabetes, or organ transplant, mechanical ventilation, and indwelling catheter), and suspected case of esophageal candidiasis can be diagnosed.

Suspected cases of esophageal candidiasis should be treated with short-term fluconazole antifungal therapy. Esophageal candidiasis can be diagnosed when symptoms recover after fluconazole treatment. In these cases, no further investigation is required. If the infection persists, further investigation may be required and the patient will then conduct the following investigation.

#### 5.2.1. Endoscopy

**Figure 1:**
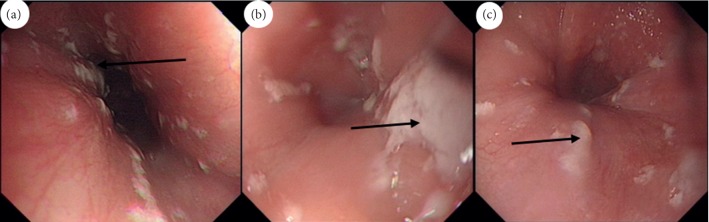
Esophageal candidiasis. Endoscopic finding; multiple whitish plaques (black arrows) are seen and are usually taken for histology and microscopic examination on brush.

Esophagoscopy is the diagnosis of choice for candida esophagitis. Direct visualization of the esophageal mucosa confirms the presence of white plaques or exudates that are adherent to the mucosa and cannot be washed off with water irrigation (Figure 1). Sometimes there may be mucosal breaks or ulcerations [17].

#### 5.2.2. Histology

The next step is to identify the source of these white plaques. The gold standard for the diagnosis of candida esophagus is by histological examination. Biopsy or brushing of the esophageal mucosa is taken during endoscopy, and staining by using hematoxylin and eosin is done. Candida yeast is almost always shown as pseudohyphae, which is an important basis for the diagnosis of esophageal candidiasis. The mucous membrane involved may present as desquamated parakeratosis, characterized by a group of squamous cells that have detached or are in the process of separating from the main squamous epithelium [11].

#### 5.2.3. Radiological Examination

**Figure 2:**
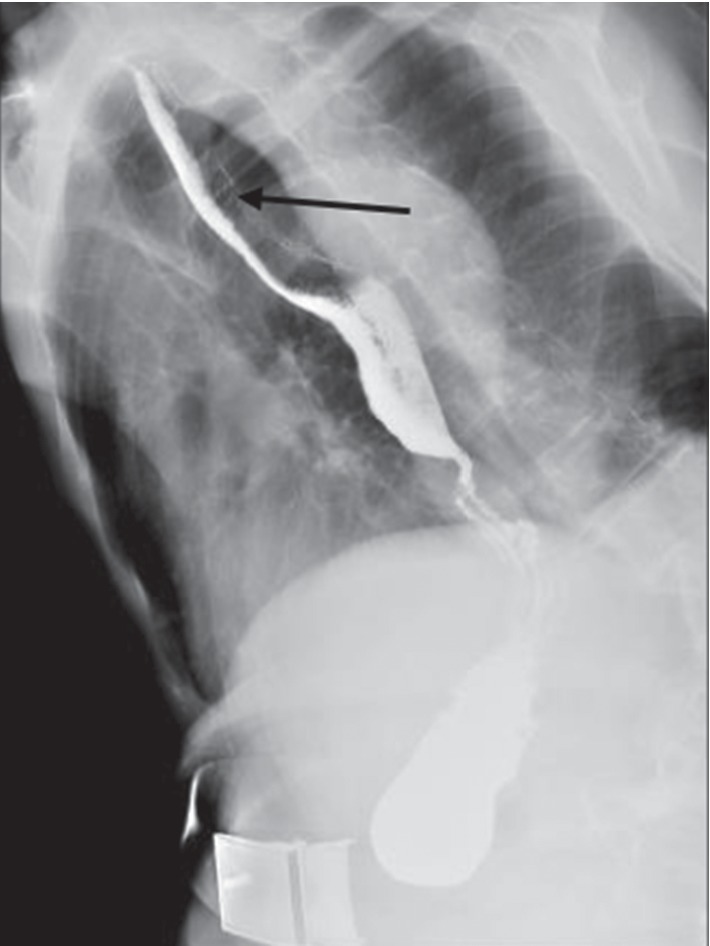
Yellow arrow indicates the characteristic “feathery” appearance of the esophageal lumen. Note that the lumen appears narrower at the area of infection while the blackish area is the fungal-infected parts.

According to Kodsi et al. [18], the disease was divided into 4 stages according to the extent of damage to the esophageal mucosa, and lumen stenosis would appear in the 4th stage. In stage 4, barium examination is a very useful noninvasive strategy for the diagnosis of candida esophagitis and can be used as an alternative to endoscopic examination. Barium swallow esophagogram presents the characteristic manifestations of esophageal stenosis, and some authors present esophageal stenosis as “foamy appearance” and “feather appearance” (Figure 2) [19–21]. Therefore, in these cases, double-contrast esophagography is a highly sensitive alternative to the diagnosis of candida esophagitis. Reports show that the sensitivity of double-contrast esophagoscopy to endoscopic diagnosis of candida esophagitis is up to 90% [4, 22].

## 6. Differential Diagnosis

Although infectious esophagitis is very common, especially *Candida albicans*, other forms of esophagitis are also prevalent. The trend and frequency differ based on the cause, susceptibility, and geographic area. Other causes include cytomegalovirus [23], herpes simplex virus, eosinophilic esophagitis, [24, 25] pill-induced esophagitis, gastroesophageal reflux disease, radioactive esophagitis, or any other form of esophageal mucosal inflammation [9, 23].

## 7. Treatment

Esophageal candidiasis usually responds well to antifungal therapy. In contrast to oropharyngeal candidiasis, the treatment of esophageal candidiasis is usually systemic rather than topical. The most commonly used medication for the treatment of esophageal candidiasis is the systemic antifungal with oral fluconazole 200 to 400 mg per day for 14 to 21 days [26]. For patients who may not be able to tolerate oral medication, the alternative is 400 mg of fluconazole intravenously daily. Itraconazole 200 mg per day orally or voriconazole 200 mg twice daily for 14 to 21 days are other treatment options. Amphotericin B deoxycholate 0.3 to 0.7 mg/kg per day may also be used in patients with nonresponsive candida esophagitis, but it has serious medication side effects, and clinicians should avoid routine use. Treatment with posaconazole 400 mg twice a day orally for patients with severe and refractory esophageal candidiasis appears to be significantly efficient [1, 27].

Other health-related conditions affect the choice of medication. For example, amphotericin B can be used for esophageal candidiasis during pregnancy in the first trimester, as teratogenic azole compounds are contraindicated [28]. Treatment with azole antifungal drugs for esophageal candidiasis rarely leads to significant side effects, but the most common symptoms include abdominal pain, nausea, vomiting, and diarrhea.

Besides active and effective antifungal therapy, dehydration, electrolyte disturbance, and acidosis should be corrected in time. It is also necessary to improve patients' general condition, improve the immune function of the body, strengthen nutrition, actively treat basic diseases, and control blood sugar. Minimize or discontinue the use of broad-spectrum antimicrobial agents and immunosuppressants. The combined use of intestinal flora regulator and intestinal mucosal protection drugs can improve the efficacy, and the application of B vitamins can enhance the resistance of local tissues and inhibit the growth of candida.

## 8. Antifungal Drug Resistance

Fluconazole is still considered as a first-line agent in EC patients with no other contraindications. However, there have been noted that frequent clinical relapses and increased antifungal utilization for prophylaxis reason which are linked to increased risks of antifungal resistance, particularly fluconazole. [29]. In the randomized clinical studies conducted previously, evidences suggest that overuse of fluconazole or other antifungal agents increases the risk of drug resistance because of dosed- dependent sensitivity [30, 31]. Patients experiencing fluconazole-refractory esophageal candidiasis (B-II) should be treated with itraconazole solution (200 mg/day Po), voriconazole (200 mg B.I.D), or caspofungin (50 mg/day) (A-II). Or intravenous amphotericin B deoxycholate (0.3–0.7 mg/kg/day) [1, 32] can be considered.

## 9. Prognosis

Few investigators have studied the prognostic sequelae of esophageal candidiasis. Usually, EC responds successfully with antifungal agents. Resistant and refractory infections may occur and may require alternative agents for treatment or long-term antifungal prophylaxis to reduce recurrence [33].

## 10. Complications

Usually, esophageal candidiasis occurs in the form of superficial esophagitis. Few cases of transmural necrosis candidiasis have been reported and are associated with serious immunosuppression and neutropenia [34]or other comorbid conditions such as patients on hemodialysis [35]. The recovery of these patients is a critical concern because the mortality rate is high.

### 10.1. Necrotizing Esophageal Candidiasis

This is the common and entry source of the rest complications. Esophageal ulcerations predispose to esophageal perforation and upper gastrointestinal bleeding, weight loss, malnourishment, sepsis, candidemia, and fistula formation into a bronchial tree [36].

### 10.2. Esophageal Stricture

Stricture to the esophagus may occur especially if the candida esophageal infection is accompanied by other conditions such as connective tissue disease or glycogen storage disease [37] or those without other underlying diseases [20].

## 11. Conclusion

Esophageal candidiasis remains one of the most common and challenging infections of the esophagus, especially in patients with low immune function and who use spectrum antibiotics and proton-pump inhibitors. Esophageal endoscopy and histological examination can accurately diagnose the disease. For patients with difficulties in endoscopic examination, barium swallow esophagogram can also be used as an auxiliary diagnosis. In clinical practice, the pretreatment evaluation model is usually used to make diagnostic decisions. In terms of treatment, oral empirical treatment with the first line of systemic antifungal is enough. However, in severe cases, prompt investigation and aggressive treatment, such as intravenous antifungal therapy, are necessary.
